# Granular Cell Tumor: A Mimicker of Breast Carcinoma

**DOI:** 10.5334/jbsr.2409

**Published:** 2021-04-05

**Authors:** Frederik Bosmans, Sofie Dekeyzer, Filip Vanhoenacker

**Affiliations:** 1 AZ Sint-Maarten, BE; 2 University (Hospital) Antwerp/Ghent, BE

**Keywords:** breast, granular cell tumor, ultrasound, MRI, mammography

## Abstract

**Teaching point:** Granular cell tumors are rare soft tissue tumors that may occur in the breast. While almost always benign, they may mimic a malignant tumor both clinically and on imaging.

## Case

**Figure 1:**
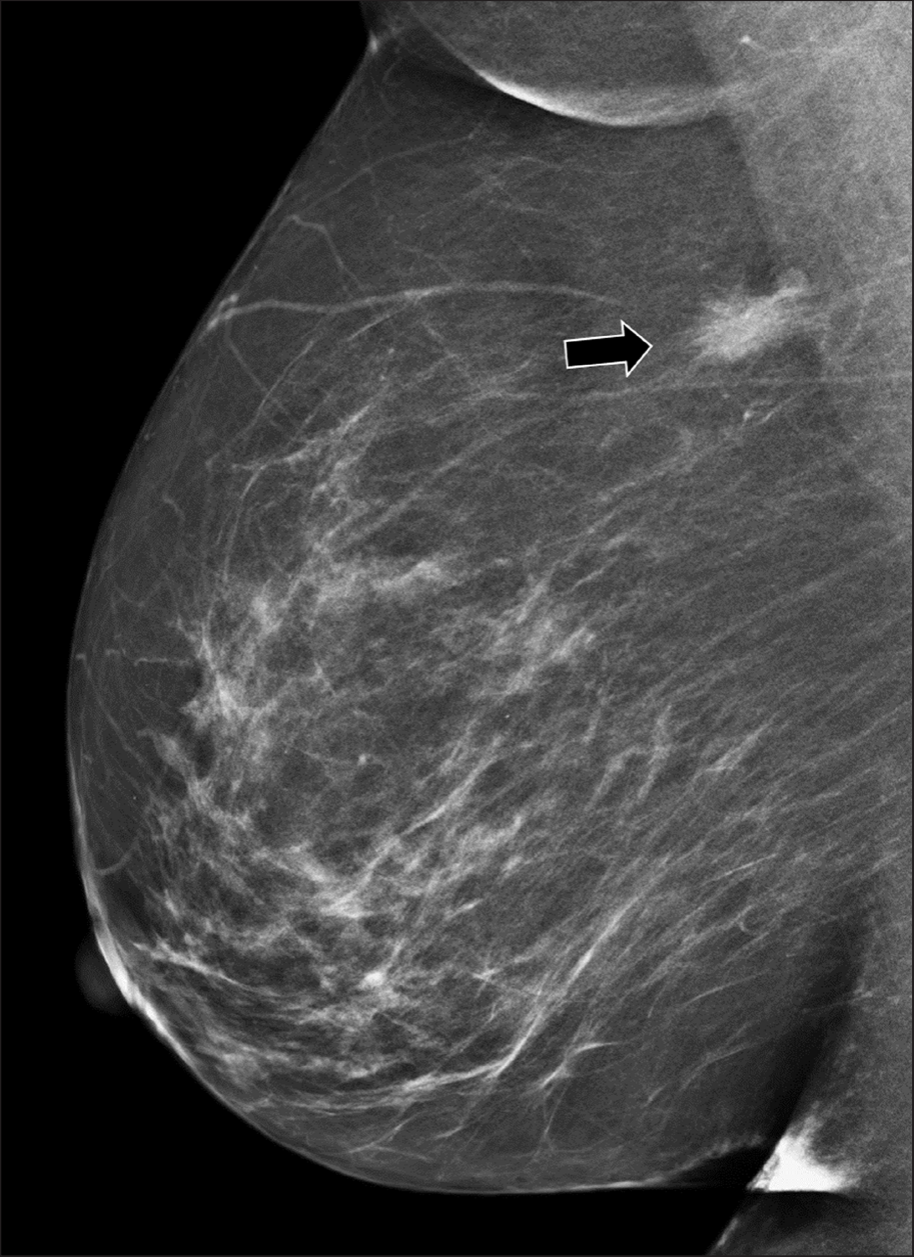


**Figure 2:**
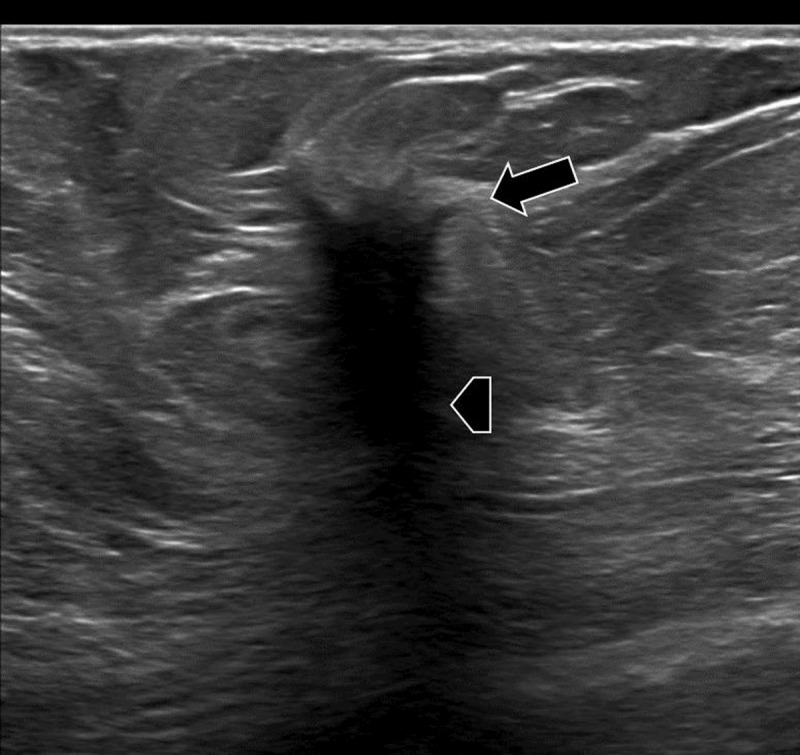


**Figure 3:**
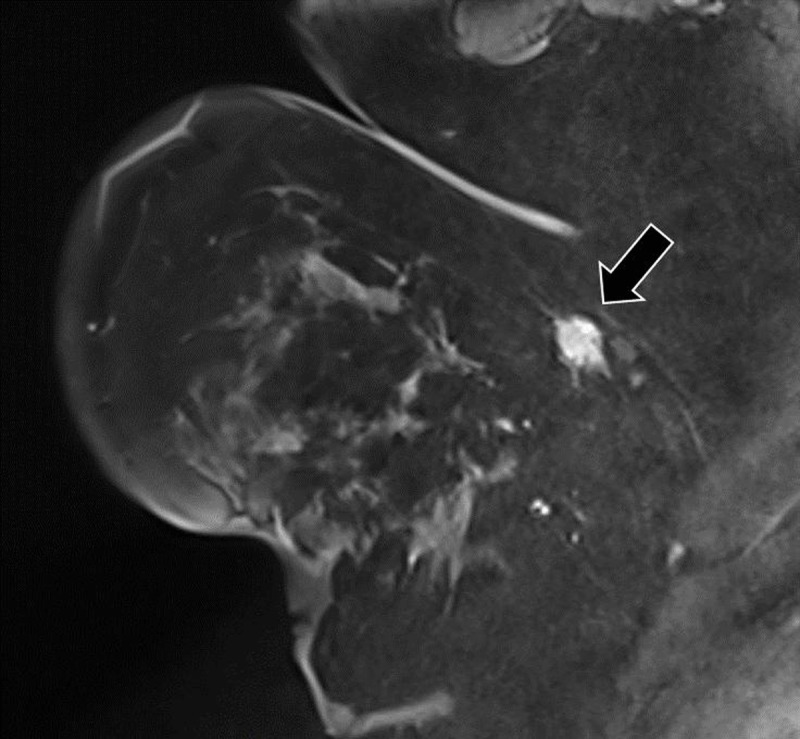


Routine breast screening examination in an asymptomatic 61-year-old female patient revealed a suspicious lesion in the axillary tail of the right breast. There was no history of breast cancer. Mammography showed a dense, spiculated mass at the upper outer quadrant of right left breast (***Figure 1***, arrow). Ultrasound demonstrated an irregular delineated hypoechoic lesion. There was a subtle partial hyperechogenic halo (***Figure 2***, arrow) and marked posterior acoustic shadowing (***Figure 2***, arrowhead). No pathological axillary lymph nodes were found. On magnetic resonance imaging (MRI), the lesion had spicular margins and avid, homogenous contrast enhancement on T1-weighted images (WI) (***Figure 3***, arrow). Histopathology confirmed the diagnosis of a granular cell tumor of the breast.

## Comment

Granular cell tumors (GrCT) are rare tumors of neural origin with Schwannian differentiation. Typically, a GrCT has abundant granular eosinophilic cytoplasm on microscopy, from which the tumor derives its name. A GrCT can occur in all soft tissues and has a prevalence of 6.7:1000 in the population undergoing evaluation for breast cancer. While mostly benign, malignancy has been reported in 0.5%–2.0% of cases.

Clinically, a GrCT is a solitary and painless mass that may be identified on palpation. On mammography, a GrCT can present as a benign-looking, well-defined nodule or show features suggestive of malignancy such as irregular margins, spiculation, and architectural distortion. Calcifications are generally absent.

Similar to mammography, on ultrasound a GrCT can appear as a well-circumscribed solid nodule or as an ill-defined heterogenic mass with variable vascularization. Sometimes a hyperechogenic halo may be seen, and posterior acoustic shadowing may be present depending upon the degree of reactive fibrosis. The most specific feature is the presence of anisotropy. A degree of variable echogenicity, depending on the angle of the insonating beam, may be seen due to the internal fibrillary composition. On MRI, a GrCT is of low to intermediate signal intensity on T1-WI but is often inconspicuous on T2-WI. Enhancement is variable after administration of gadolinium contrast. Both progressive (type 1) and wash-out (type 3) dynamic curves have been described. Additionally, a GrCT does not have increased metabolic activity on PET-CT; this feature can be helpful to differentiate a GrCT from malignancy.

Due to the non-specific imaging findings, tissue analysis is required for definite diagnosis.

While the prognosis of a GrCT is generally good, metastatic disease has been described. The recommended treatment is wide surgical excision considering the higher risk of recurrence with positive resection margins [1].
